# Metabolic reprogramming enables the auxiliary diagnosis of breast cancer by automated breast volume scanner

**DOI:** 10.3389/fonc.2022.939606

**Published:** 2022-10-12

**Authors:** Jianjun Liu, Yang Zhou, Huiying Liu, Mengyan Ma, Fei Wang, Chang Liu, Qihang Yuan, Hongjiang Wang, Xiukun Hou, Peiyuan Yin

**Affiliations:** ^1^ Clinical Laboratory of Integrative Medicine, The First Affiliated Hospital of Dalian Medical University, Dalian, China; ^2^ College of Integrative Medicine, Dalian Medical University, Dalian, China; ^3^ Department of Ultrasound, The First Affiliated Hospital of Dalian Medical University, Dalian, China; ^4^ Breast Surgery, The First Affiliated Hospital of Dalian Medical University, Dalian, China; ^5^ Department of General Surgery, The First Affiliated Hospital of Dalian Medical University, Dalian, China

**Keywords:** metabolomics, breast cancer, automated breast volume scanner, metabolites, biomarker

## Abstract

Breast cancer is the leading cause of female cancer-related deaths worldwide. New technologies with enhanced sensitivity and specificity for early diagnosis and monitoring of postoperative recurrence are in critical demand. Automatic breast full volume scanning system (ABVS) is an emerging technology used as an alternative imaging method for breast cancer screening. Despite its improved detection rate of malignant tumors, ABVS cannot accurately stage breast cancer preoperatively in 30–40% of cases. As a major hallmark of breast cancer, the characteristic metabolic reprogramming may provide potential biomarkers as an auxiliary method for ABVS.

**Objective:**

The objective of this study was to identify differential metabolomic signatures between benign and malignant breast tumors and among different subtypes of breast cancer patients based on untargeted metabolomics and improve breast cancer detection rate by combining key metabolites and ABVS.

**Methods:**

Untargeted metabolomics approach was used to profile serum samples from 70 patients with different subtypes of breast cancer and benign breast tumor to determine specific metabolomic profiles through univariate and multivariate statistical data analysis.

**Results:**

Metabolic profiles correctly distinguished benign and malignant breast tumors patients, and a total of 791 metabolites were identified. There were 54 different metabolites between benign and malignant breast tumors and 17 different metabolites between invasive and non-invasive breast cancer. Notably, the missed diagnosis rate of ABVS could be reduced by differential metabolite analysis. Moreover, the diagnostic performance analyses of combined metabolites (pelargonic acid, *N*-acetylasparagine, and cysteine-S-sulfate) with ABVS performance gave a ROC area under the curve of 0.967 (95% CI: 0.926, 0.993).

**Conclusions:**

Our study identified metabolic features both in benign and malignant breast tumors and in invasive and non-invasive breast cancer. Combined ultrasound ABVS and a panel of differential serum metabolites could further improve the accuracy of preoperative diagnosis of breast cancer and guide surgical therapy.

## Introduction

Breast cancer (BC) is the most commonly diagnosed cancer and one of the leading causes of cancer death among women worldwide (1). So, it badly threatened the female’s health, and the occurrence showed a young trend. In 2012, nearly 1.7 million women were diagnosed with the disease, making it a global priority (2). The reliable and timely diagnosis of BC can significantly affect treatment. There are diverse diagnostic techniques for detecting and categorizing BC, for example, ultrasound (US), X-ray mammography, magnetic resonance imaging (MRI), and biopsy. The current gold standard in BC diagnosis is biopsy. However, this technique is invasive, time consuming, and can yield some negative results to some extent (3). In addition, BC is a biologically variable disease with different subtypes, showing different biological behavior and response to treatment and prognosis. Thus, non-invasive, fast, sensible, and precise methods for early diagnosis and distinguishing different BC subtypes are in critical demand.

Automatic breast full volume scanning system (ABVS) is one of the latest technological breakthroughs that have been proposed as a suitable alternative for BC screening (4). It is a safe, painless, radiation-free, and non-invasive technology. It is a three-dimensional volume imaging system that can provide data from the entire breast. ABVS has some advantages, including non-radioactivity, sensitivity to dense breast, three-dimensional reconstruction, time saving, and repeatability (5). Although ABVS has improved the detection rate of malignant tumors, it still cannot accurately stage BC preoperatively in 30–40% of cases (3). Therefore, can it be combined with other bio-molecules to improve the accuracy of BC diagnosis?

Metabolomics provides changes in metabolites in biological systems in response to pathophysiological stimuli or genetic variation (6). Metabolomics has been widely used as a promising strategy for the identification of disease markers, which might be an auxiliary method for ABVS. As the final downstream products of gene transcription, metabolites are closely linked to biological functions and phenotypes, so they could offer important insights into disease mechanisms and identify potential diagnostic or prognostic biomarkers (7, 8). For breast tumors, regardless of benign, malignant, or multiple subtypes of BC, it implies the uniqueness of the interactions between tumor and a specific patient population, which could lead to distinct metabolic changes in each group (9). Herein, we designed this study to identify differential metabolomic signatures between benign and malignant breast tumors and among different subtypes of BC patients based on untargeted metabolomics and to explore whether metabolomics can be an auxiliary method for ABVS to add early diagnosis information in individuals with breast neoplasm.

## Materials and methods

### Reagents and solutions

Methanol, acetonitrile, isopropanol, formic acid, and ammonium acetate were purchased from Thermo Fisher Scientific (Fair Lawn, NJ). Ammonium bicarbonate (LC-MS grade) and methyl tert-butyl ether (MTBE) were purchased from Sigma-Aldrich (St. Louis, MO). Ultra-pure water (18.2 m ω cm) was prepared in house by a Milli-Q purified water system (Merck KGaA, Darmstadt, Germany).

### Participants and ethics

A total of 70 patients with benign and malignant tumors of breast were enrolled from July 2019 to July 2020 at the breast surgery of the First Affiliated Hospital of Dalian Medical University. All subjects included 21 women with benign breast diseases (BE) and 49 women with malignant breast diseases (BC). Diagnoses in the benign breast diseases were 17 with fibroadenoma and four with papilloma. Malignant breast diseases included 38 with invasive carcinoma (28 with non-specific invasive carcinoma, five with invasive lobular carcinoma, and five with papillary carcinoma) and 11 with non-invasive carcinoma (intraductal carcinoma). The ABVS examination was carried out on all the patients. The preoperative ABVS diagnosis contained eight false negative (two with nonspecific invasive carcinoma and six with intraductal carcinoma) and three false positive (three with a papilloma), which were used as the validation set. The criteria for selection included at last 18 years old with histological confirmation of patients with benign and malignant breast tumor, no detectable macro metastatic disease and no prior anticancer treatment in BC group, and none of the patients with benign breast diseases had any malignancy diseases in their past history. Exclusion criteria: Subjects without ABVS tests or BC patients with malignant metastasis diseases were excluded. The demographic characteristics and clinical diagnosis of these subjects are summarized in **Table 1**. This study was approved by the Ethics Committee of the First Affiliated Hospital of Dalian Medical University (PJ-KS-KY-2019-78), and informed consent was obtained from all participants.

**Table 1:** Demographic and clinical pathological characteristics of study population.

Parameters	Malignant group	Benign group
	(*n* = 49)	(*n* = 21)
Age (median, range)	56(30–75)	48(19–82)
Fibroadenoma, *n* (%)	n.a.	17(81.0)
Papilloma, *n* (%)	n.a.	4(19.0)
Non-specific invasive carcinoma, *n* (%)	28(57.1)	n.a.
Invasive lobular carcinoma, *n* (%)	5(10.2)	n.a.
Papillary carcinoma, *n* (%)	5(10.2)	n.a.
Intraductal carcinoma, *n* (%)	11(22.4)	n.a.
TNM stage-0	8(16.3)	n.a.
TNM stage-Ia	23(46.9)	n.a.
TNM stage-IIa	11(22.4)	n.a.
TNM stage-IIb	3(6.1)	n.a.
TNM stage-IIIa	3(6.1)	n.a.
TNM stage-IIIc	1(2)	n.a.
ABVS (0)*	8 (false negative)	18
ABVS (1)*	41	3(false positive)

*Resulting visibility values dichotomized “0” and “1” for ABVS (“0” represents no abnormality and “1” represents abnormal).

### Automated Breast Volume Scanner

It was performed using Acuson S2000 Automated Breast Volume Scanner (ABVS; Siemens, Munich, Germany), a computer-based system for evaluating the whole breast, with the patient in a supine position. First, two-dimensional hand-held Doppler US was used to check the size, location, nature, boundary, shape, and blood supplement of the mass, and then a technician maintained appropriate contact pressure and vertical orientation to the breast surface during ABVS examination. After full scanning of all sections, it is transmitted to the computer for data processing to form three-dimensional images. The results were interpreted by two senior US experts. It is divided into no abnormality and abnormality (including angulation, burr, calcification, and aggregation), which had a malignant tendency.

### Serum sample preparation and processing

The blood samples were collected in anticoagulant-free blood collection tubes in the morning after at least 8h of fasting before surgery. All patients had not received any medication, anesthetic, or other therapy. The samples were centrifuged at 1500*g* for 10 min at 4°C after standing for 1h at 4°C to obtain the serum. The serum samples were isolated, aliquoted, and immediately stored at -80°C until further use. Take 150 μl of each serum sample and place it in the 96-Deep Well plates (Thermo Fisher Scientific, Delaware, USA). Then, add 600 μl of polar extract (mixture of methanol and acetonitrile) to the sample. After that, swirl the mixture for 5 min and centrifuge it at 5300 RPM for 20 min (4°C). After centrifugation, two doses of 200 μl of supernatant were transferred to the 96-well plates (Thermo Fisher Scientific). Then, the samples were concentrated and dried in a vacuum lyophilizer. The 50% methanol was added to these two plates and redissolved, followed by positive and negative ion detection and subsequent untargeted metabolomics analysis. Mix the remaining top layers of all remaining samples; take 150 μl from each of them as quality control (QC) samples.

### Untargeted metabolomic analysis

An Ultimate 3000 ultra-high performance liquid chromatography (UHPLC) and Q Exactive Quadrupole-Orbitrap High-Resolution Mass Spectrometer (HRMS; Thermo Fisher Scientific) were used for untargeted metabolomics analysis. The metabolite extracts were profiled with reversed-phase chromatographic separation mode with positive and negative ionization detection, respectively. For the positive detection mode, an ACE C18-PFP column (1.8 μm, 2.1 × 100 mm; ACE Co., Leicestershire, United Kingdom) was used and eluted by 0.1% formic acid in water as mobile phase A and acetonitrile as mobile phase B. A linear gradient was used ramping from 2% organic mobile phase to 98% in 10 min. For the negative detection mode, the mobile phases A and B that contain 400-mg ammonium bicarbonate buffer salt was used to elute metabolites and separated on an Acquity HSS C18 column (Waters Corporation, Milford, MA, 1.8 μm, 2.1 mm × 100 mm). The mobile phase gradient was as follows: phase B from 0 min 2% ramped to 100% in 10 min and followed by 5 min of column washing and equilibration. The flow sampling volume and column temperature of positive and negative modes were the same, which were 0.4 ml/min 5 μl 50°C, respectively.

Metabolites were detected by using a heated electrospray source, and the same ionization parameters were set except ionization voltage, including 45 arb of sheath gas and 10 arb of aux gas, heater temperature to 355°C, capillary temperature to 320°C, and S-Lens RF level to 55%. The metabolomic extracts were analyzed with full scan mode under 70,000 FWHM resolution with AGC 1 × 10^6^ and 200 ms max injection time by using a scan range of 70–1,000 m z^−1^ to obtain data.

### Data processing

There are secondary annotations that need to be paid attention to according to the recommendations of the Metabolomics Standardization Initiative (MSI) (10). First, the chromatographic retention time and primary and secondary mass spectrometry information should be consistent with the standard. The second is to annotate the structure of polar metabolites by searching against a local library created using authentic standards as well as mzCloud library (Thermo Fisher Scientific, San Jose, CA). In addition, m/z of MS1 spectra was searched against a local HMDB metabolite chemical database (11). Mass accuracy of precursor within ±5 ppm was a prerequisite for metabolite identification or structural annotation. The area under curve values as extracted as quantitative information of polar metabolites with TraceFinder software (Thermo Fisher Scientific).

### Statistical analysis

The metabolomic data from different measurements were normalized and merged. Variables were deleted with missing value percentages above 50% and then input the missing values with the K-nearest algorithm (KNN sample wise). The metabolites detected by various methods are retained only once to ensure their uniqueness of the metabolites. Sample calibration, data conversion and data scaling are three steps in the normalization of untargeted metabolomics data. First, sample calibration corrected sample reproducibility during testing due to batch effects or systematic errors. Next, in order to convert the data to a normal distribution, we performed Log transformations on the untargeted metabolomics data. Finally, the potential structure discriminant analysis (PLS-DA) data were preprocessed by orthogonal projection using UV scaling transformation. Multivariate analysis, such as the PLS-DA and orthogonal PLS-DA (OPLS-DA) were conducted with SIMCA-P 14.1 software (metrics, Sweden). Univariate analysis including independent sample *t*-test and the false discovery rate (FDR; adjusted *p* < 0.05) and fold change (FC; adjusted *p* < 0.05) adjusting with Benjaminiand Hochberg method and a heat map was drawn to perform on the MetaboAnalyst website (http://www.metaboanalyst.ca). We applied the R software package “neuralnet” to an artificial neural network model of the differentially expressed metabolites (DEMs; *p* < 0.05). First, the calculation of model ber was based on the premise of out-of-band data. Set 57 to the optimal number of trees contained in the random forest. Next, the decreasing accuracy method (Gini coefficient method) was used to obtain the dimension importance value of the random forest model on the premise of constructing the random forest model first. Then, the top three metabolites with the highest AUC area were selected for subsequent modeling. We used the R software package “neuralnet” to construct an artificial neural network model of the essential variables after the normalization of the data. We set five hidden layers for the model parameters and then use the obtained metabolite weight information to construct the breast-cancer-classification model. The sum of the product of weight score and the metabolite expression level was used as the disease classification score to evaluate the diagnostic performance of the model. The pROC software package was used to demonstrate the performance of the neural network model more intuitively (12). In addition, we used GraphPad Prism 8.0 Software (GraphPad Software Inc.) to plot receiver operating characteristic (ROC) curves and calculated 95% confidence interval (95% CI) cutoff values for the area under the curve (AUC), and plotted volcanoes to find highly varied metabolites, and then box plots were drawn to describe the specific metabolites that differ between the two groups. For the combined indicators, logistic regression analysis was based on SPSS Statistics 26.0 software (IBM).

## Results

### Study design and clinical characteristics

**Figure 1:**
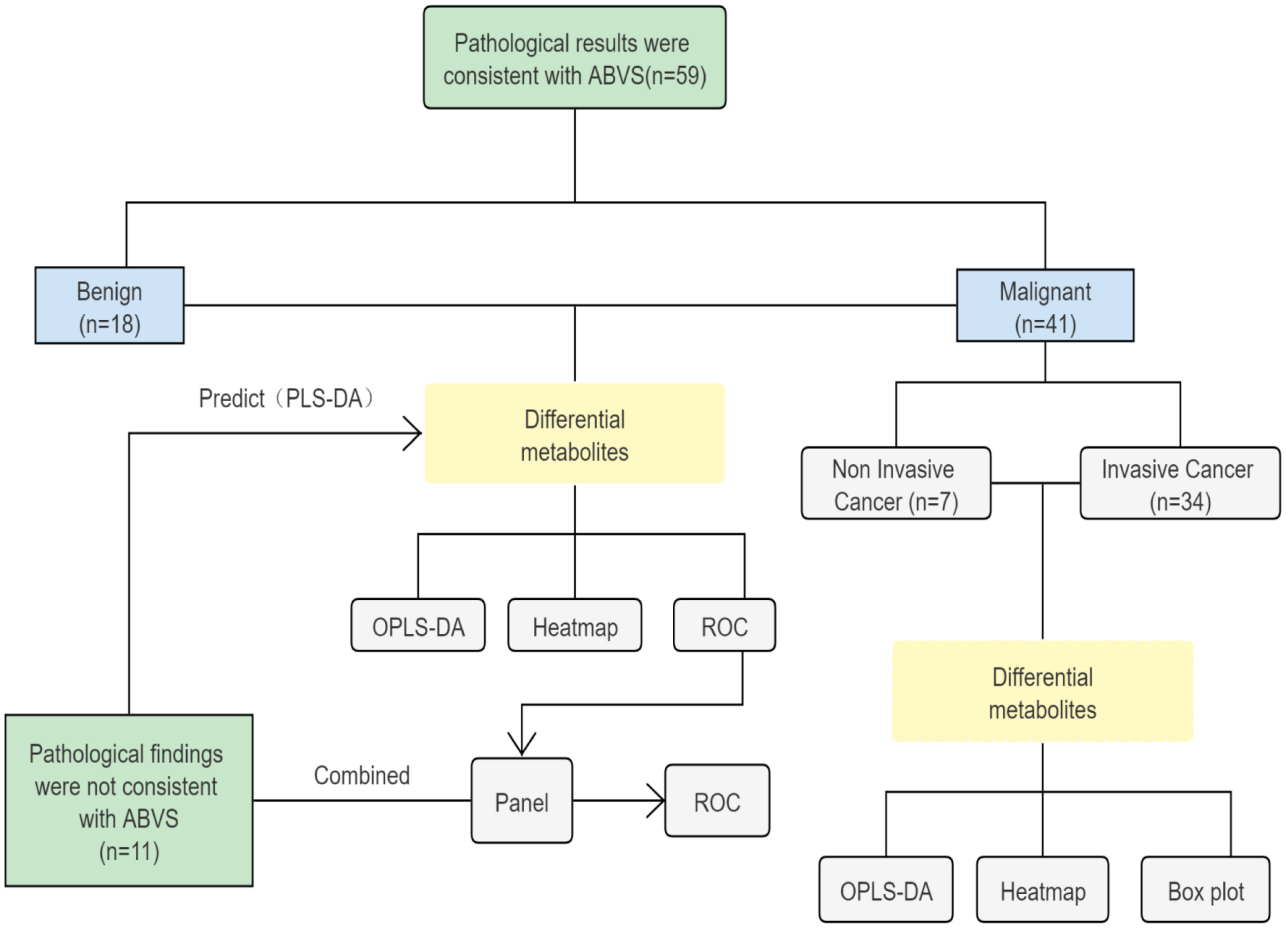
The design and workflow of this study.

A workflow of the study is shown in **Figure 1**. First, we tried to characterize the molecular alterations in the serum of patients with benign and malignant breast lesions. Second is to identify differential metabolomic signatures in invasive and non-invasive breast cancer. Finally, combined with ABVS examination and key metabolites to improve the detection rate of preoperative breast malignant tumors. Sera from 49 malignant and 21 benign breast lesions were enrolled. Details of the inclusion and exclusion criteria are described in the part of the method. The clinical features of the participants are shown in **Table 1**.

### Metabolic features of benign and malignant breast lesions

**Figure 2:**
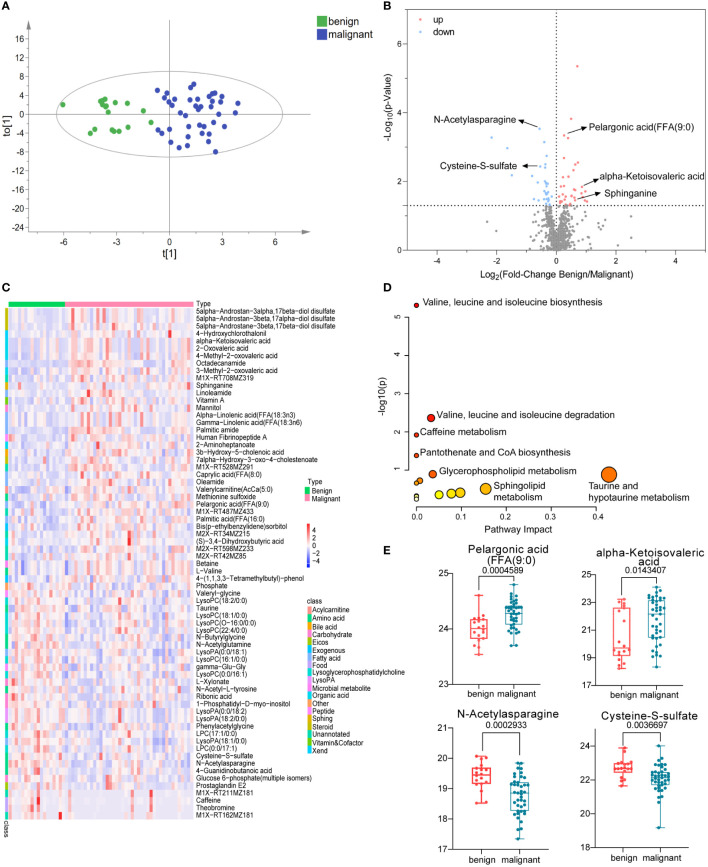
Polar metabolites in benign and malignant breast tumor group. OPLS-DA score plots of metabolites between benign and malignant group **(A)**. The volcano plots of metabolites, the red dots of the metabolites in the volcano plots indicated an increase and the blue dots indicate a decrease in the malignant group **(B)**. The relative concentration of metabolites in each sample in benign and malignant group was visualized using a heat map **(C)**. Metabolic pathway enrichment analysis based on KEGG database was performed to determine differentially enriched pathways between benign and malignant group **(D)**. Relative concentration of pelargonic acid (FFA (9:0), alpha-ketoisovaleric acid, *N*-Acetylasparagine, and cysteine S-sulfate in benign and malignant group **(E)**.

Untargeted metabolomics was employed to describe the characteristics of serum metabolism among patients with benign and malignant breast lesions. A total of 791 metabolites (786 metabolites remaining after data screening and cleaning) were identified. The coefficient of variation (CV) distribution of QC, which was used to demonstrate the reproducibility of the method, was shown in **Supplementary Figure S1**. Polar metabolite OPLS-DA (**Figure 2A**) was drawn to illustrate metabolic changes between the benign and malignant groups, from which we observed an overall separation between the two groups. The volcano map (**Figure 2B**) showed the levels of expression of compounds between the two groups. There were 28 upregulated metabolites, such as pelargonic acid, FFA (9:0), alpha-ketoisovaleric acid, sphinganine, and so on whereas 26 downregulated metabolites, such as *N*-Acetylasparagine, Cysteine-S-sulfate, and so on (**Supplementary Table S1**). These were enriched in seven pathways (**Figure 2D**); the most significant of which were the biosynthesis and degradation of valine, leucine and isoleucine, taurine and hypotaurine metabolism, and the sphingolipid metabolism. A heat map visually exhibited the relative concentration differences of these metabolites between the two groups (FDR-adjusted *p* =< 0.05, **Figure 2C**). The differentially represented metabolites was shown in the boxplot graph (**Figures 2E**), including fatty acids (e.g., pelargonic acid, FFA 9:0, FC = 1.2, *p* < 0.01), organic acid (e.g., alpha-ketoisovaleric acid, FC =1.81, *p* < 0.05), and amino acids (e.g., *N*-Acetylasparagine, FC = 0.68, *P* < 0.001 and cysteine S-sulfate, FC = 0.69, *P* < 0.01). The detailed list of metabolite differences was shown in **Supplementary Table S1**. The above results illustrated that significantly altered fatty acids, amino acids, organic acids, and so forth might be related to the pathogenesis of BC.

### Metabolic features of invasive and non-invasive breast cancer

**Figure 3:**
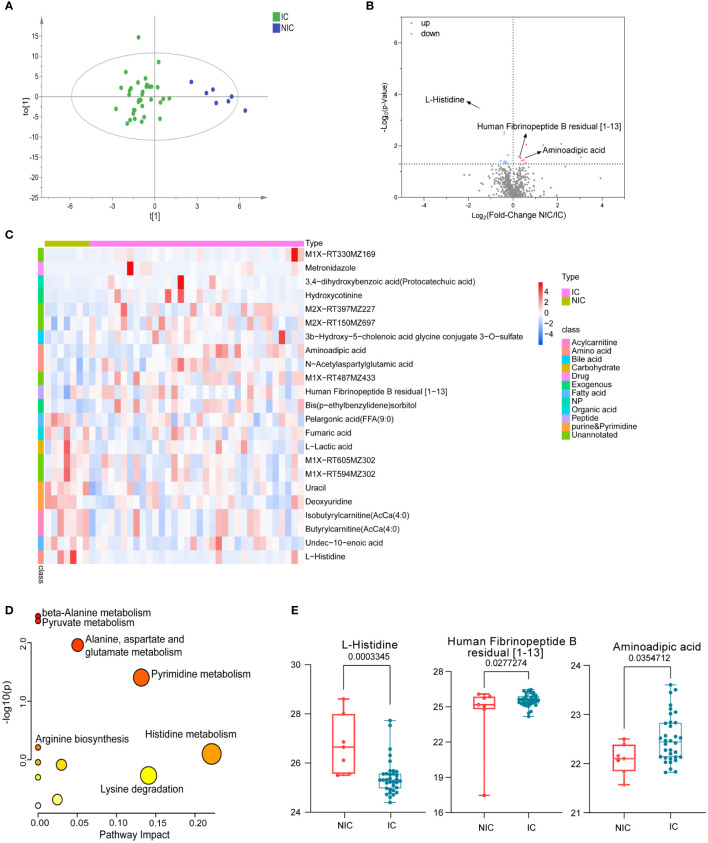
Polar metabolites in non-invasive (NIC) and invasive breast cancer (IC) group. OPLS-DA score plots of metabolites between non-invasive and invasive groups **(A)**. Volcano plots of metabolites. The red dots of the metabolites in the volcano plots indicated an increase, and the blue dots indicate a decrease in the invasive cancer group **(B)**. Relative concentration of metabolites in each sample in non-invasive and invasive group was visualized using a heat map **(C)**. Metabolic pathway enrichment analysis based on KEGG database was performed to determine differentially enriched pathways between non-invasive and invasive groups **(D)**. Relative concentration of L-Histidine, aminoadipic acid, and Human Fibrinopeptide B residual in the non-invasive and the invasive cancer group **(E)**.

According to the pathological classification of breast cancer, we further divided the malignant group into non-invasive and invasive cancer. Similarly, OPLS-DA was used to illustrate metabolic changes between the two groups (**Figure 3A**). The results suggested that there were significant differences between the two groups. Significantly, differential polar metabolites in invasive and non-invasive BC were visualized on volcano plots (**Figure 3B**). Among them, there were eight upregulated and nine downregulated metabolites in the invasive group compared to the non-invasive group (**Supplementary Table S2**), which also caused changes in metabolic pathways (**Figure 3D**), including histidine metabolism, lysine biosynthesis, pyrimidine metabolism, alanine, aspartate, and glutamate metabolism and so on. A heat map was used to visualize the relative concentration differences of metabolites in the two groups (FDR-adjusted *p* =< 0.05, **Figure 3C**). The expression levels of key metabolites in the two groups were represented by box plots (**Figure 3E**), the levels of aminoadipic acid (FC = 1.39, *p* < 0.05) and Human Fibrinopeptide B residual (FC = 1.25, *p* < 0.05) increased obviously, while L-Histidine (FC = 0.35, *p* < 0.01) decreased in patients with invasive BC comparing with BE. They were involved in lysine biosynthesis, fibrinogen oligopeptide degradation, and histidine metabolism, respectively, which probably are associated with tumor metastasis.

### Distinguish the cases whose ABVS examination was inconsistent with postoperative pathological diagnosis based on metabolomics

**Figure 4:**
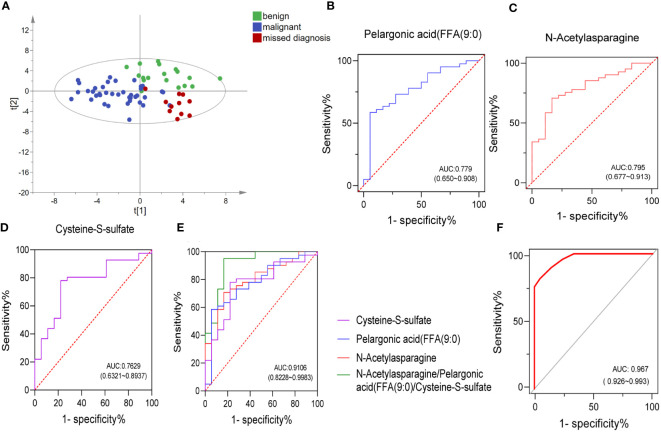
Combining a panel of metabolites and ABVS examination to improve the efficacy of discriminating the malignant breast tumors from benign tumors. OPLS-DA score plots of metabolites among benign, malignant, and ABVS missed diagnosis group **(A)**. Receiver-operating characteristic (ROC) curves of the three biomarkers and their combination **(B–E)**. ROC curve analysis was used to intuitively show the effectiveness of the model (combing a panel of metabolites and ABVS performance) in distinguishing benign and malignant breast tumors **(F)**.

Although ABVS has improved the detection rate of malignant breast tumors and shows some advantages, it still has the possibility of missed diagnosis (false negative) and misdiagnosis (false positive). In our enrolled subjects, three cases returned false positive (misdiagnosing a benign patient as having cancer) and eight cases returned false negative (missing the malignant tumor as it spreads). Toward to these 11 patients, OPLS-DA analysis was performed to detect whether these cohort, including false negative and false positive cases, could be recognized correctly based on the previous differential metabolites between benign and malignant breast tumors. Fortunately, the results showed that only one missed case was located between benign and malignant groups. The remaining seven cases were correctly distributed in the malignant group; however, the other three misdiagnosed cases were not identified and existed in the malignant group (**Figure 4A**). Nevertheless, the detection of metabolites greatly recognized malignant cases, which could provide a reference for clinicians and reduce the missed diagnosis rate.

### Combining a panel of metabolites and ABVS examination to improve the efficacy of discriminating the malignant breast tumors from benign tumors

The observed separation tendencies in our multivariate approach indicated the possibility of compiling a panel of metabolites to discriminate the malignant breast tumors from benign tumors. An artificial neural network model of combing a panel of metabolites and ABVS performance was constructed, and a receiver operator characteristic (ROC) curve analysis was used to intuitively show the effectiveness of the model in distinguishing benign and malignant breast tumors. ROC curves for the significantly altered metabolites were drawn to distinguish the benign and malignant groups (**Supplemental Table S3**). The top 3 metabolites with the highest AUC area were *N*-acetylasparagine, pelargonic acid (FFA[9:0]) and cysteine-S-sulfate. The levels of these molecules changed significantly in malignant group relative to benign group. The level of pelargonic acid, FFA(9:0), was much higher in malignant than in benign tumors, with AUC values of 0.779 (95% CI: 0.650, 0.908) (**Figures 2E**, **4B**). The serum levels of *N*-acetylasparagine and cysteine-S-sulfate (**Figure 2E**) were descended in malignant relative to benign tumors with AUC values of 0.796 (95% CI: 0.677, 0.913, **Figure 4C**) and 0.763 (95% CI: 0.632, 0.894, **Figure 4D**), respectively. The AUC value of the three metabolites combined was 0.911 (95% CI: 0.823, 0.998, **Figure 4E**). Notably, the AUCs were greatly improved when combining these three metabolites with ABVS performance (AUC = 0.967, 95% CI: 0.926, 0.993, **Figure 4F**). The results highlighted that combining with the serum differential metabolites could improve the efficacy of ABVS to discriminate the malignant breast tumors from benign tumors.

## Discussion

As in most malignant diseases, early breast cancer detection is crucial for effective diagnosis and treatment, enhanced patient survival, and reduced death rate (13). Although, ABVS has improved the detection rate of malignant tumors, a certain proportion of cases that cannot be accurately identified before operation (3). Therefore, it is necessary to develop novel biomarkers to auxiliary differentiate benign and malignant breast tumors and improve the accuracy of diagnosis. Breast cancer has been associated with marked metabolic shifts (14, 15), and metabolomics has been widely applied to refine molecular sub-typing of breast cancer, cancer progression, cancer metastasis, and prediction of treatment sensitivity (16–18). This study identified metabolic reprogramming both in benign and malignant breast tumors and in invasive and non-invasive breast cancer. In addition, to verify whether the metabolites can correctly distinguish the cases that cannot be recognized by ABVS. Our study tested the hypothesis of an improvement in the diagnostic sensitivity of breast cancer using candidate metabolites and ABVS.

Some significantly changed metabolites were identified from the samples, which may be related to tumorigenesis. First, pelargonic acid, FFA(9:0); nonylic acid; and pelargic acid. Callol-Sanchez et al. found a significantly elevated level of nonanoic acid in exhaled breath of patients with lung cancer compared with chronic obstructive pulmonary disease (COPD) patients and healthy subjects (19). In addition, it was also reported that the nonanoic acid was increased in patients with oral cancer (20) and prostate cancer (21). Another study showed that nonanoic acid (C9:0), which belongs to odd-chain fatty acids, is present in trace levels in human tissue. It was reported that odd-chain fatty acids acted as histone deacetylases (HDACs) inhibitors, whereas the dysregulation of HDACs is closely associated with tumorigenesis (22). Moreover, they also found that this kind of odd-chain fatty acids could promote the acetylation of α-tubulin in MCF-7 breast and A549 lung cancer cells dose-dependently and had moderate anti-proliferative effects (22). In our study, the serum pelargonic acid, FFA(9:0), was elevated in breast cancer patients with an AUC of 0.78 (**Figure 4B**). The specific mechanism needs to be further studied. Interestingly, we also found that the levels of some acetylated metabolites decreased significantly, such as *N*-acetylasparagine (FC = 0.68, *P* < 0.001, **Figure 2E**) with a higher AUC of 0.795 (**Figure 4C**), *N*-acetyl-l-tyrosine (FC = 0.79, *P* < 0.05) and *N*-acetylglutamine (FC = 0.81, *P* < 0.05). It might be related to the disorder of enzymes associated with acetylation and deacetylation.

In addition, alpha-ketoisovaleric acid (KIV), a kind of branched-chain α-keto acids (BCKAs), is metabolized from branched-chain amino acids—valine. This reaction is catalyzed by branched-chain aminotransferase (BCAT) (23). In this study, KIV was significantly elevated in breast cancer (**Figure 2E**), and it was involved in valine, leucine, and isoleucine biosynthesis and degradation, which had been shown significant changes in metabolic pathway analysis (**Figure 2D**). BCAAs are not synthesized from BCKAs in humans as essential amino acids. However, in myeloid leukemia cells, BCAT is intensively expressed and promotes a reverse reaction to synthesize BCAAs from BCKAs (24). Other studies have shown that BCAT is related to breast cancer, non-small cell lung cancer, ovarian cancer and liver cancer (25–28). Therefore, we speculated the mutual transformation between KIV and BACC may be involved in the pathogenesis of breast cancer.

Moreover, we found that sphinganine was increased in patients with breast cancer compared with benign tumors (FC = 1.54, *p* < 0.05, **Supplementary Figure S2**). It is also elevated in other cancers, such as pancreatic cancer (29) and endometrial cancer (30). Sphinganine is an intermediate of sphingoid base biosynthesis (31, 32). Upregulation of sphinganine suggests that sphingolipid metabolism is hampered in cancer progression (29), which has been confirmed in our metabolic pathway analysis (**Figure 2D**). Sphinganine can be catalyzesd by Sphingosine kinase 1 (SPHK1) to generate sphingosine-1-phosphate (S1P) (33), which has played an important role in regulating the death and survival of cancer cells (34, 35). In addition, sphingosine kinase 1 (SPHK1) was overexpressed in triple-negative breast cancer and promoted metastasis *via* nuclear factor kappa B/sphingosine kinase 1 (NFκB/SPHK1) signaling pathway activation (36). Therefore, sphinganine might implicate in the initiation and progression of breast cancer.

Last, we also paid attention to the significant decline of two metabolites in breast cancer. One is cysteine S-sulfate, which is a glutamate receptor agonist that can lead to calcium influx in nerve cells and neurotoxicity when present at high levels (37, 38). A previous investigation reported the heritability of plasma cysteine S-sulfate to be 46.8%, suggesting that both genetic and environmental factors strongly influence it. A recent study showed that lower levels of the amino acid cysteine S-sulfate was associated with poorer executive function with increasing age (39), whereas high levels of cysteine S-sulfate may be detrimental to cognitive function earlier in life (39). In this study, we also found that the level of cysteine S-sulfate descended in the serum of patients with breast cancer compared with benign tumors (FC = 0.69, *p* < 0.01, **Figure 2E**), with an AUC of 0.763 (**Figure 4D**). However, further study will be crucial to understand the mechanisms by which cysteine S-sulfate could have protective effects on breast cancer.

Taurine, a β-amino acid produced by the liver, is distributed in various tissues at high concentrations and helps in maintaining the functions of the central nervous system, retinal neurons, heart, and skeletal muscles. Increasing evidence has shown that exogenous taurine has the anti-tumor activity against different cancers *in vitro* and *in vivo* (40), such as breast cancer (41, 42), colorectal cancer (43), and lung cancer (44). The mechanism of taurine inhibiting the growth and metastasis of breast cancer involves multiple targets and pathways (41). Another research found that serum antioxidant taurine in breast cancer group exhibited a significantly lower level than that in the control group. The results suggested that taurine had the potential to be a novel tumor marker for enhanced detection of breast cancer in the early diagnosis (45). Coincidentally, we also found decreased content of serum taurine in breast cancer patients (FC = 0.84, *p* < 0.05, **Supplementary Table S1**), which may weaken its protective effect and promote tumorigenesis.

Among invasive and non-invasive breast cancer patients, we found some metabolites with significant differences, such as L-histidine, human fibrinopeptide B, and aminoadipic acid. It is reported that L-histidine could play a role in preventing or suppressing tumor development (46). However, it decreased obviously in the invasive group (FC = 0.35, *p* < 0.01, **Figure 3E**). Human fibrinopeptides A and B were released from fibrinogen during blood coagulation. Research showed that fibrinogen levels were significantly elevated in breast cancer than that in the control group, and metastatic patients exhibited significantly higher D-dimer values when compared with early breast cancer patients (47), which is consistent with our results. In this study, human fibrinopeptides A and B were significantly increased in the malignant group (**Supplementary Figure S2**) and invasive breast cancer (**Figure 3E**), respectively. Moreover, aminoadipic acid, a product of lysine degradation, increased in patients with invasive breast cancer (**Figure 3E**). It has also been found elevated in chemotherapy recipients after 6 months (48) and suggested as a predictive biomarker for the development of diabetes (49). Nevertheless, multiple studies have shown that lactate and lactate dehydrogenase(LDH)may play a role in the progression of breast cancer, especially LDH. A meta-analysis showed that serum LDH could act as a diagnostic factor for patients with breast cancer (50). It has been reported that the concentration of lactic acid in serum and tumor tissue of breast cancer patients is increased (51, 52), but it is still controversial. This study found that the serum lactate level of non-invasive patients was higher than that of invasive patients. The specific mechanism needs to be further studied by expanding the sample size.

Breast cancer is a heterogeneous disease consisting of distinct histopathological subtypes with different clinical outcomes (53). There is a certain probability of missed diagnosis(false negative)and misdiagnosis(false positive)in ABVS detection. Among the samples that we enrolled, there were eight false negative (two with nonspecific invasive carcinoma and six with intraductal carcinoma) and three false positive (three with a papilloma). Among the false negative, six cases were intraductal carcinoma. This could be attributed to the fact that the tumor was located in the mammary duct, so ABVS has no characteristic manifestations. Takayoshi also confirmed some breast tumors such as ductal carcinoma *in situ* and invasive lobular carcinoma were easily missed on US because of the nature of the lesions (54). While metabolomics might make up for this defect and could identify these patients (**Figure 4A**). Three false positives were all papilloma. The main manifestations of papilloma and papillary carcinoma were unclear boundaries from ABVS detection and hard to be distinguished, which might be due to the situation that fibrosis at the edge of papillomas often entraps glands and creates the spurious impression of invasion (55). In this study, however, it also could not be well distinguished based on metabolomics. A larger sample size of papilloma and papillary carcinoma might be conducive to distinguish them. Conclusively, detection of metabolites greatly recognized malignant cases from benign and reduce the misdiagnosis rate of ABVS.

Although our study provided original insights into the metabolic reprogramming enabled the auxiliary diagnosis of breast cancer by AVBS, there were still some limitations. First, the sample size was relatively small and, therefore, no further hierarchical analysis was performed for different molecular subtypes. Second, although metabolomic testing can greatly improve the missed diagnosis rate of ABVS, it is better to be validated in a larger cohort. Finally, this study aimed to improve breast cancer detection rate by combining key metabolites and ABVS. If more other clinical indicators could be added for correlation analysis, it would be benefit to validate the metabolomic results.

## Conclusions

Our study tested the hypothesis of an improvement in the diagnostic sensitivity of breast cancer by combining key metabolites (pelargonic acid, *N*-acetylasparagine, and cysteine-S-sulfate) and ABVS examination. The missed diagnosis rate of ABVS was obviously reduced by differential metabolite analysis. This study indicated that noninvasive ABVS examination and potential biomarkers derived from characteristic metabolic reprogramming could provide doctors and patients with more accurate and valuable diagnostic references before operation.

## Data availability statement

The original contributions presented in the study are included in the article/**Supplementary Material**. Further inquiries can be directed to the corresponding authors.

## Ethics statement

The studies involving human participants were reviewed and approved by the Ethics Committee of the First Affiliated Hospital of Dalian Medical University. The patients/participants provided their written informed consent to participate in this study.

## Author contributions

PY, XH and JL designed the study and managed the study. JL, HL, MM and CL conducted the experimental work. JL, YZ, MM and FW collected healthy and patient samples, HW and FW provided clinical and scientific expertise to this project. YZ and XH did ABVS test and interpreted the results. HL, QY and JL conducted the data analysis. JL, HL, PY and XH wrote and edited the paper. All authors contributed to the article and approved the submitted version.

## Funding

This work was supported by the National Natural Science Foundation of China (no. 82004152), Liaoning Provincial Natural Science Foundation of China (2019-MS-082).

## Conflict of interest

The authors declare that the research was conducted in the absence of any commercial or financial relationships that could be construed as a potential conflict of interest.

## Publisher’s note

All claims expressed in this article are solely those of the authors and do not necessarily represent those of their affiliated organizations, or those of the publisher, the editors and the reviewers. Any product that may be evaluated in this article, or claim that may be made by its manufacturer, is not guaranteed or endorsed by the publisher.
