# Supplementary material for: Metabolic reprogramming enables the auxiliary diagnosis of breast cancer by automated breast volume scanner
Source: Front Oncol. 2022 Oct 12;12:939606. doi: 10.3389/fonc.2022.939606 (PMC9597368; doi:10.3389/fonc.2022.939606)
Supplement: Supplementary file 1 [file DataSheet_1.doc]

**Supplementary figure S1.** **RSD% distribution in QCs samples.**


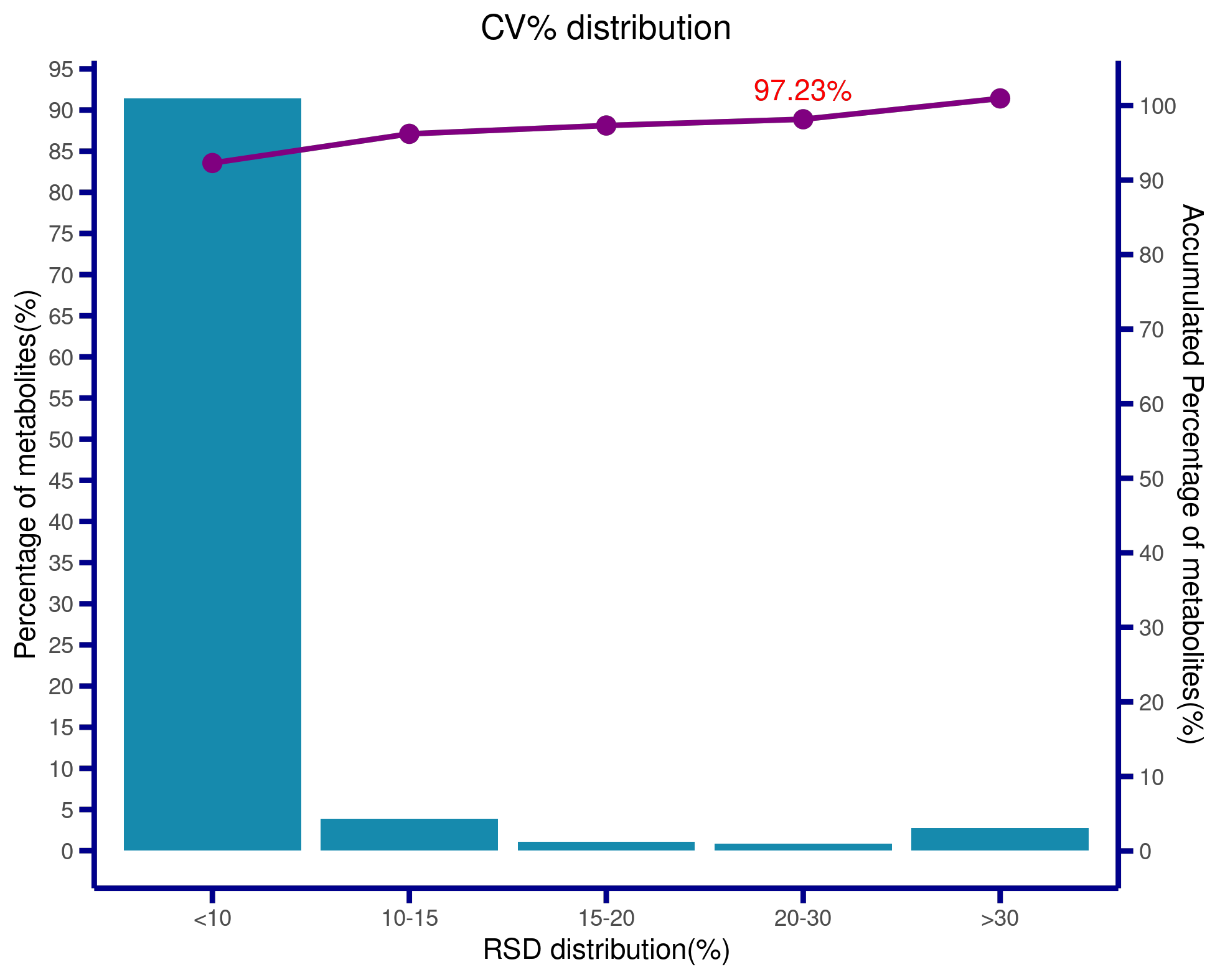


**Supplementary figure S2. Changes of human fibrinopeptide A and sphinganine in benign and malignant groups.**


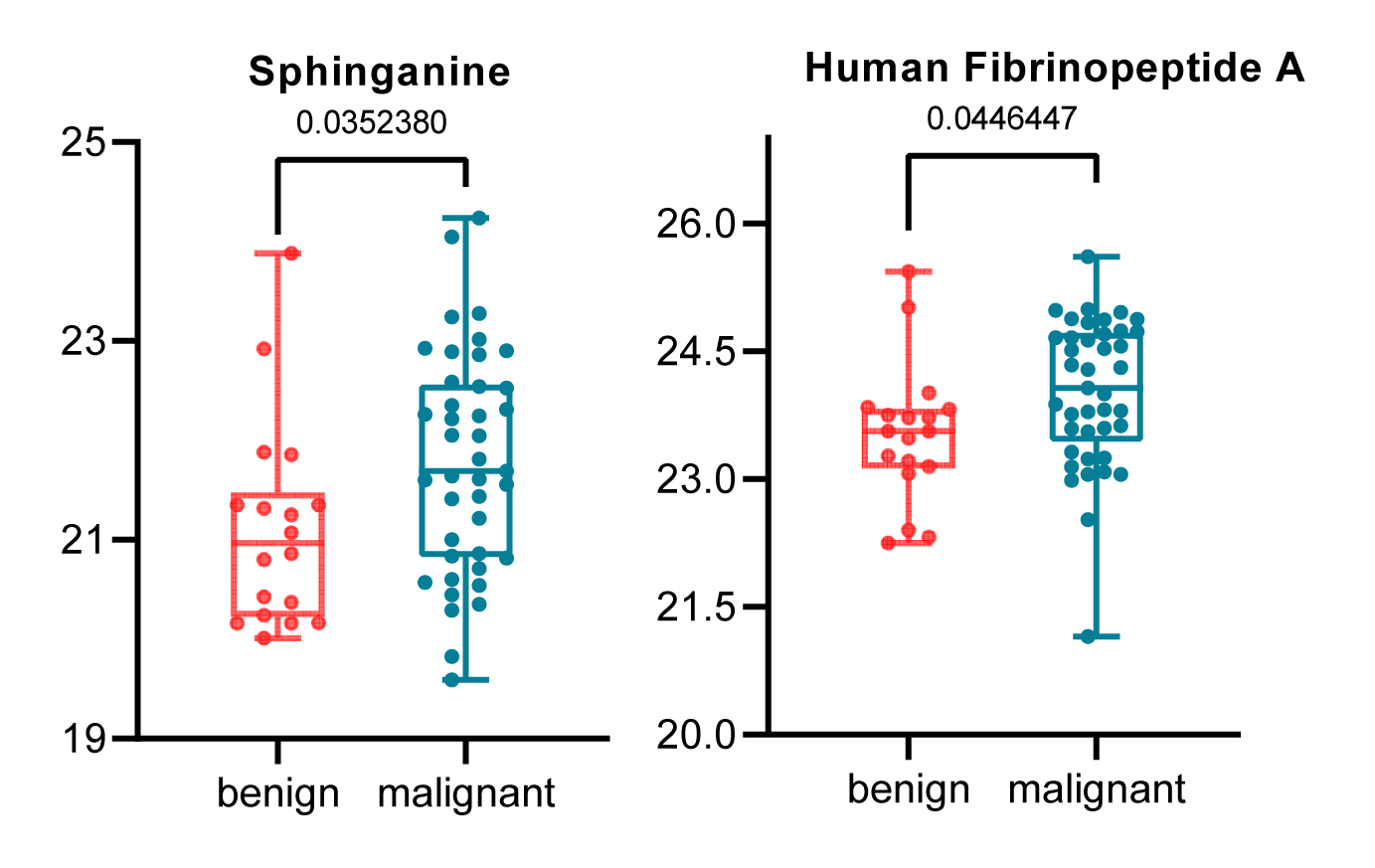


**Supplementary Table S1. Differential metabolites in benign and malignant groups**

| Class | Metabolites | Log2(FC) | Fold change | Log10  (P-value) | P-value |
| --- | --- | --- | --- | --- | --- |
| Steroid | 5alpha-Androstan-3alpha,17beta-diol disulfate | 1.0276 | 2.0387 | 1.4160 | 3.84E-02 |
| Steroid | 5alpha-Androstan-3beta,17alpha-diol disulfate disulfate | 0.9714 | 1.9607 | 1.7118 | 1.94E-02 |
| Steroid | 5alpha-Androstane-3beta,17beta-diol disulfate | 0.9553 | 1.9389 | 1.4490 | 3.56E-02 |
| Organic acid | alpha-Ketoisovaleric acid | 0.8577 | 1.8121 | 1.8434 | 1.43E-02 |
| Organic acid | 2-Oxovaleric acid | 0.8411 | 1.7915 | 1.6537 | 2.22E-02 |
| Organic acid | 4-Methyl-2-oxovaleric acid | 0.7254 | 1.6534 | 2.5520 | 2.81E-03 |
| Fatty acid | Octadecanamide | 0.7032 | 1.6281 | 5.3533 | 4.43E-06 |
| Organic acid | 3-Methyl-2-oxovaleric acid | 0.6348 | 1.5527 | 2.4942 | 3.20E-03 |
| Sphing | Sphinganine | 0.6276 | 1.5450 | 1.4530 | 3.52E-02 |
| Fatty acid | Linoleamide | 0.6036 | 1.5195 | 1.5300 | 2.95E-02 |
| Vitamin&Cofactor | Vitamin A | 0.5903 | 1.5056 | 1.7671 | 1.71E-02 |
| Carbohydrate | Mannitol | 0.5733 | 1.4879 | 2.3274 | 4.71E-03 |
| Fatty acid | Alpha-Linolenic acid(FFA(18:3n3) | 0.5081 | 1.4222 | 1.5844 | 2.60E-02 |
| Fatty acid | Gamma-Linolenic acid(FFA(18:3n6) | 0.5063 | 1.4204 | 1.5745 | 2.66E-02 |
| Fatty acid | Palmitic amide | 0.4930 | 1.4073 | 3.8238 | 1.50E-04 |
| Peptide | Human Fibrinopeptide A | 0.4665 | 1.3817 | 1.3502 | 4.46E-02 |
| Organic acid | 2-Aminoheptanoate | 0.4420 | 1.3585 | 1.3068 | 4.93E-02 |
| Bile acid | 3b-Hydroxy-5-cholenoic acid | 0.4075 | 1.3264 | 2.1399 | 7.25E-03 |
| Steroid | 7alpha-Hydroxy-3-oxo-4-cholestenoate | 0.4036 | 1.3228 | 2.1431 | 7.19E-03 |
| Fatty acid | Caprylic acid(FFA(8:0) | 0.3130 | 1.2423 | 1.5598 | 2.76E-02 |
| Fatty acid | Oleamide | 0.3097 | 1.2395 | 1.5581 | 2.77E-02 |
| Acylcarnitine | Valerylcarnitine(AcCa(5:0) | 0.3085 | 1.2384 | 1.3251 | 4.73E-02 |
| Amino acid | Methionine sulfoxide | 0.2789 | 1.2133 | 1.4713 | 3.38E-02 |
| Fatty acid | Pelargonic acid(FFA(9:0) | 0.2605 | 1.1979 | 3.3383 | 4.59E-04 |
| Fatty acid | Palmitic acid(FFA(16:0) | 0.2553 | 1.1936 | 2.6836 | 2.07E-03 |
| Organic acid | (S)-3,4-Dihydroxybutyric acid | 0.1801 | 1.1330 | 1.4190 | 3.81E-02 |
| Microbial metabolite | Betaine | 0.1235 | 1.0893 | 1.4011 | 3.97E-02 |
| Amino acid | L-Valine | 0.1222 | 1.0884 | 1.3800 | 4.17E-02 |
| Peptide | Valeryl-glycine | -0.2206 | 0.8582 | 1.3035 | 4.97E-02 |
| Lysoglycerophosphatidylcholine | LysoPC(18:2/0:0) | -0.2288 | 0.8533 | 1.3273 | 4.71E-02 |
| Amino acid | Taurine | -0.2522 | 0.8396 | 1.4619 | 3.45E-02 |
| Lysoglycerophosphatidylcholine | LysoPC(18:1/0:0) | -0.2676 | 0.8307 | 1.5078 | 3.11E-02 |
| Lysoglycerophosphatidylcholine | LysoPC(O-16:0/0:0) | -0.2742 | 0.8269 | 1.6914 | 2.04E-02 |
| Lysoglycerophosphatidylcholine | LysoPC(22:4/0:0) | -0.2748 | 0.8266 | 1.4248 | 3.76E-02 |
| Amino acid | N-Butyrylglycine | -0.2889 | 0.8185 | 1.9332 | 1.17E-02 |
| Amino acid | N-Acetylglutamine | -0.3113 | 0.8059 | 1.6396 | 2.29E-02 |
| Fatty acid | LysoPA(0:0/18:1) | -0.3248 | 0.7984 | 2.7446 | 1.80E-03 |
| Lysoglycerophosphatidylcholine | LysoPC(16:1/0:0) | -0.3277 | 0.7968 | 1.4860 | 3.27E-02 |
| Peptide | gamma-Glu-Gly | -0.3278 | 0.7968 | 1.8607 | 1.38E-02 |
| Lysoglycerophosphatidylcholine | LysoPC(0:0/16:1) | -0.3382 | 0.7910 | 1.6247 | 2.37E-02 |
| Carbohydrate | L-Xylonate | -0.3383 | 0.7910 | 1.9552 | 1.11E-02 |
| Amino acid | N-Acetyl-L-tyrosine | -0.3459 | 0.7868 | 1.3525 | 4.44E-02 |
| Carbohydrate | Ribonic acid | -0.3477 | 0.7858 | 1.9391 | 1.15E-02 |
| Carbohydrate | 1-Phosphatidyl-D-myo-inositol | -0.3553 | 0.7817 | 1.6634 | 2.17E-02 |
| LysoPA | LysoPA(0:0/18:2) | -0.3661 | 0.7759 | 2.4994 | 3.17E-03 |
| LysoPA | LysoPA(18:2/0:0) | -0.3758 | 0.7707 | 2.4037 | 3.95E-03 |
| Amino acid | Phenylacetylglycine | -0.3838 | 0.7664 | 2.0048 | 9.89E-03 |
| Lysoglycerophosphatidylcholine | LPC(17:1/0:0) | -0.4010 | 0.7573 | 1.7211 | 1.90E-02 |
| Fatty acid | LysoPA(18:1/0:0) | -0.4047 | 0.7554 | 3.1448 | 7.16E-04 |
| Lysoglycerophosphatidylcholine | LPC(0:0/17:1) | -0.4141 | 0.7505 | 1.4836 | 3.28E-02 |
| Amino acid | Cysteine-S-sulfate | -0.5410 | 0.6873 | 2.4354 | 3.67E-03 |
| Amino acid | N-Acetylasparagine | -0.5644 | 0.6762 | 3.5327 | 2.93E-04 |
| Amino acid | 4-Guanidinobutanoic acid | -0.5981 | 0.6606 | 1.4498 | 3.55E-02 |
| Carbohydrate | Glucose 6-phosphate(multiple isomers) | -0.6220 | 0.6498 | 1.9715 | 1.07E-02 |

**Supplementary Table S2. Differential metabolites in non-invasive and invasive breast cancer groups**

| Class | Metabolites | Log2(FC) | Fold change | Log10  (P-value) | P-value |
| --- | --- | --- | --- | --- | --- |
| Bile acid | 3b-Hydroxy-5-cholenoic acid glycine conjugate 3-O-sulfate | 0.5491 | 1.4632 | 1.3419 | 4.55E-02 |
| Amino acid | Aminoadipic acid | 0.4774 | 1.3923 | 1.4501 | 3.55E-02 |
| Amino acid | N-Acetylaspartylglutamic acid | 0.3826 | 1.3037 | 1.4229 | 3.78E-02 |
| Peptide | Human Fibrinopeptide B residual [1-13] | 0.3245 | 1.2523 | 1.5571 | 2.77E-02 |
| Fatty acid | Pelargonic acid(FFA(9:0) | -0.2334 | 0.8506 | 1.6403 | 2.29E-02 |
| Organic acid | Fumaric acid | -0.2741 | 0.8270 | 1.3312 | 4.66E-02 |
| Carbohydrate | L-Lactic acid | -0.3246 | 0.7985 | 1.3984 | 4.00E-02 |
| purine&Pyrimidine | Uracil | -0.3962 | 0.7599 | 2.5313 | 2.94E-03 |
| purine&Pyrimidine | Deoxyuridine | -0.4009 | 0.7574 | 2.4467 | 3.58E-03 |
| Acylcarnitine | Isobutyrylcarnitine(AcCa(4:0) | -0.4060 | 0.7547 | 1.3303 | 4.67E-02 |
| Acylcarnitine | Butyrylcarnitine(AcCa(4:0) | -0.4143 | 0.7504 | 1.3524 | 4.44E-02 |
| Fatty acid | Undec-10-enoic acid | -0.5554 | 0.6805 | 1.4140 | 3.85E-02 |
| Amino acid | L-Histidine | -1.5278 | 0.3468 | 3.4756 | 3.35E-04 |

**Supplementary Table S3. AUC values of differential metabolites in benign and malignant groups**

| Name | AUC | Name | AUC | Name | AUC |
| --- | --- | --- | --- | --- | --- |
| N-Acetylasparagine | 0.7954 | Pelargonic acid(FFA(9:0) | 0.7791 | Cysteine-S-sulfate | 0.7629 |
| Theobromine | 0.7507 | 3b-Hydroxy-5-cholenoic acid | 0.7426 | 5alpha-Androstan-3alpha,17beta-diol disulfate | 0.7385 |
| Bis(p-ethylbenzylidene)sorbitol | 0.7358 | 7alpha-Hydroxy-3-oxo-4-cholestenoate | 0.7331 | Mannitol | 0.7317 |
| LysoPA(0:0/18:2) | 0.7317 | 5alpha-Androstan-3beta,17alpha-diol disulfate disulfate | 0.7290 | 5alpha-Androstane-3beta,17beta-diol disulfate | 0.7290 |
| alpha-Ketoisovaleric acid | 0.6978 | 3-Methyl-2-oxovaleric acid | 0.7222 | Caffeine | 0.7222 |
| LysoPA(18:2/0:0) | 0.7209 | 4-Methyl-2-oxovaleric acid | 0.7168 | (S)-3,4-Dihydroxybutyric acid | 0.7100 |
| 2-Aminoheptanoate | 0.7073 | Vitamin A | 0.7046 | LysoPC(18:2/0:0) | 0.7046 |
| Glucose 6-phosphate(multiple isomers) | 0.7046 | LysoPC(18:1/0:0) | 0.7019 | N-Acetylglutamine | 0.7019 |
| alpha-Ketoisovaleric acid | 0.6978 | Sphinganine | 0.6938 | Human Fibrinopeptide A | 0.6734 |
